# Supplementary material for: A survey of gyrodactylid parasites on the fins of Homatula variegata in central China
Source: PLoS One. 2020 Mar 18;15(3):e0230320. doi: 10.1371/journal.pone.0230320 (PMC7080252; doi:10.1371/journal.pone.0230320)
Supplement: Attachment — Submitted filename: PONE-D-19-26341_reviewer.pdf [file pone.0230320.s002.pdf]

PLOS ONE

A survey of gyrodactylid parasites on the fins of Homatula variegata in central China

--Manuscript Draft--

**Manuscript Number:**	PONE-D-19-26341
**Article Type:**	Research Article
**Full Title:**	A survey of gyrodactylid parasites on the fins of Homatula variegata in central China
**Short Title:**	Chen et al. Two gyrodactylid on Homatula variegata
**Corresponding Author:**	Ping You, Ph.D.<br>Shaanxi Normal University<br>Xi'an, Shaanxi CHINA
**Keywords:**	Homatula variegata; Gyrodactylidae; Site specificity; Qinling Mountains
**Abstract:**	In this study, two parasites on the fins were recorded from March to September 2016. A dissection mirror was used to examine the distribution and quantity dynamics of the ectoparasitic fish Gyrodactylus sp. and Paragyrodactylus variegatus in the host Homatula variegata, in different seasons. The present study will explore possible explanations for the site specificity of gyrodactylidae parasites in 442 Homatula variegata infected with 4307 Gyrodactylus sp. and 1712 Paragyrodactylus variegatus. These two gyrodactylidae parasites were captured in China's Qinling Mountains with fishing nets and scorpions. The results indicated that the highest relative abundance of Gyrodactylus sp., which was numerically dominants species, appeared in fish fins during April, while the highest relative abundance of Paragyrodactylus variegatus was found in fish fins during March. The two parasite species appeared to be partitioned spatially, with Gyrodactylus sp. occurring more frequently on pectoral and pelvic fins, while P. variegatus occurred more frequently on the pectoral, pelvic and caudal fins. However, Gyrodactylus sp. appeared to occur similarly on fish of all lengths, while P. variegatus tended to occur more abundantly on smaller rather than larger fish. At lower Gyrodactylus sp. infection levels (<100), the pelvic and pectoral fins were the main locations of attachment, followed by the dorsal fin. For infections of more than 100 parasites, more Gyrodactylus sp. were located on the pectoral fin. In another aspect, for a low number of Paragyrodactylus variegatus infections (<100), the pelvic and pectoral fins were the preferred locations of attachment, followed by the caudal fin. Between the months of April and September, there were many monogenean parasites on the fish fins, and the fish size was within the range of 5-10 cm. However, when a fish is greater than 10cm long, the number of parasites on its fins is greatly reduced.
**Order of Authors:**	Xiaoning Chen
	Biao Wang
	Jianzhen Nie
	Ping You, Ph.D.
**Additional Information:**	
**Question**	**Response**
**Financial Disclosure**<br><br>Enter a financial disclosure statement that describes the sources of funding for the work included in this submission. Review the submission guidelines for detailed requirements. View published research articles from *PLOS ONE* for specific examples.<br><br>This statement is required for submission	This study was supported by grants from the National Natural Science Foundation of China (31872203) and the Natural Science Foundation of Shaanxi Province (2017JM3014). The funders had no role in study design, data collection and analysis, decision to publish, or preparation of the manuscript.

Powered by Editorial Manager® and ProduXion Manager® from Aries Systems Corporation

and **will appear in the published article** if the submission is accepted. Please make sure it is accurate.<br><br>**Unfunded studies**<br>Enter: *The author(s) received no specific funding for this work.*<br><br>**Funded studies**<br>Enter a statement with the following details:<br>• Initials of the authors who received each award<br>• Grant numbers awarded to each author<br>• The full name of each funder<br>• URL of each funder website<br>• Did the sponsors or funders play any role in the study design, data collection and analysis, decision to publish, or preparation of the manuscript?<br>• **NO** - Include this sentence at the end of your statement: *The funders had no role in study design, data collection and analysis, decision to publish, or preparation of the manuscript.*<br>• **YES** - Specify the role(s) played.<br><br>* typeset	
**Competing Interests**<br><br>Use the instructions below to enter a competing interest statement for this submission. On behalf of all authors, disclose any competing interests that could be perceived to bias this work—acknowledging all financial support and any other relevant financial or non-financial competing interests.<br><br>This statement **will appear in the published article** if the submission is accepted. Please make sure it is accurate. View published research articles from *PLOS ONE* for specific examples.	The authors have declared that no competing interests exist.

**NO authors have competing interests**<br><br>Enter: *The authors have declared that no competing interests exist.*<br><br>**Authors with competing interests**<br><br>Enter competing interest details beginning with this statement:<br><br>*I have read the journal's policy and the authors of this manuscript have the following competing interests: [insert competing interests here]*<br><br>* typeset	
**Ethics Statement**<br><br>Enter an ethics statement for this submission. This statement is required if the study involved:<br><br>• Human participants<br>• Human specimens or tissue<br>• Vertebrate animals or cephalopods<br>• Vertebrate embryos or tissues<br>• Field research<br><br>Write "N/A" if the submission does not require an ethics statement.<br><br>General guidance is provided below. Consult the submission guidelines for detailed instructions. **Make sure that all information entered here is included in the Methods section of the manuscript.**	N/A

Powered by Editorial Manager® and ProduXion Manager® from Aries Systems Corporation

**Format for specific study types**<br><br>**Human Subject Research (involving human participants and/or tissue)**<br>• Give the name of the institutional review board or ethics committee that approved the study<br>• Include the approval number and/or a statement indicating approval of this research<br>• Indicate the form of consent obtained (written/oral) or the reason that consent was not obtained (e.g. the data were analyzed anonymously)<br><br>**Animal Research (involving vertebrate animals, embryos or tissues)**<br>• Provide the name of the Institutional Animal Care and Use Committee (IACUC) or other relevant ethics board that reviewed the study protocol, and indicate whether they approved this research or granted a formal waiver of ethical approval<br>• Include an approval number if one was obtained<br>• If the study involved *non-human primates*, add *additional details* about animal welfare and steps taken to ameliorate suffering<br>• If anesthesia, euthanasia, or any kind of animal sacrifice is part of the study, include briefly which substances and/or methods were applied<br><br>**Field Research**<br><br>Include the following details if this study involves the collection of plant, animal, or other materials from a natural setting:<br>• Field permit number<br>• Name of the institution or relevant body that granted permission	
**Data Availability**<br><br>Authors are required to make all data underlying the findings described fully available, without restriction, and from the time of publication. PLOS allows rare exceptions to address legal and ethical concerns. See the PLOS Data Policy and FAQ for detailed information.	Yes - all data are fully available without restriction

<table>
<tr><td>A Data Availability Statement describing where the data can be found is required at submission. Your answers to this question constitute the Data Availability Statement and will be published in the article, if accepted.

Important: Stating ‘data available on request from the author’ is not sufficient. If your data are only available upon request, select ‘No’ for the first question and explain your exceptional situation in the text box.

Do the authors confirm that all data underlying the findings described in their manuscript are fully available without restriction?</td><td></td></tr>
<tr><td>Describe where the data may be found in full sentences. If you are copying our sample text, replace any instances of XXX with the appropriate details.

• If the data are held or will be held in a public repository, include URLs, accession numbers or DOIs. If this information will only be available after acceptance, indicate this by ticking the box below. For example: All XXX files are available from the XXX database (accession number(s) XXX, XXX.).
• If the data are all contained within the manuscript and/or Supporting Information files, enter the following: All relevant data are within the manuscript and its Supporting Information files.
• If neither of these applies but you are able to provide details of access elsewhere, with or without limitations, please do so. For example:

Data cannot be shared publicly because of [XXX]. Data are available from the XXX Institutional Data Access / Ethics Committee (contact via XXX) for researchers who meet the criteria for access to confidential data.

The data underlying the results presented in the study are available from (include the name of the third party</td><td>All relevant data are within the manuscript and its Supporting Information files.</td></tr>
</table>

and contact information or URL).<br>• This text is appropriate if the data are owned by a third party and authors do not have permission to share the data.<br><br>* typeset	
Additional data availability information:	

Powered by Editorial Manager® and ProduXion Manager® from Aries Systems Corporation

Manuscript
Click here to access/download;Manuscript;Chen et al. Two gyrodactylid parasites on Homatula variegata.doc

A survey of gyrodactylid parasites on the fins of *Homatula variegata* in central China

Xiaoning Chen[1,2], **Biao Wang**[1], **Jianzhen Nie**[1], **Ping You**[1]*

[1] College of Life Sciences, Shaanxi Normal University, Xi'an, Shaanxi, China
[2] Shaanxi Key Laboratory for Animal Conservation, Shaanxi Institute of Zoology, Xi'an, Shaanxi, China

*Corresponding Author:
Ping You
No. 199, South Chang'an Road, Yanta District, Xi'an 710062, China
Email address: youping@snnu.edu.cn

Abstract

In this study, two parasites on the fins were recorded from March to September 2016. A dissection mirror was used to examine the distribution and quantity dynamics of the ectoparasitic fish *Gyrodactylus* sp. and *Paragyrodactylus variegatus* in the host *Homatula variegata*, in different seasons. The present study will explore possible explanations for the site specificity of gyrodactylidae parasites in 442 *Homatula variegata* infected with 4307 *Gyrodactylus* sp. and 1712 *Paragyrodactylus variegatus.* These two gyrodactylidae parasites were c[illegible]red in China's Qinling Mountains with fishing nets and scorpions. The results indicated that the highest relative abundance of *Gyrodactylus* sp., which was numerically dominants species, appeared in fish fins during April, while the highest relative abundance of *Paragyrodactylus variegatus* was found in fish fins during March. The two parasite species appeared to be partitioned spatially, with *Gyrodactylus* sp. occurring more frequently on pectoral and pelvic fins, while *P. variegatus* occurred more frequently on the pectoral, pelvic and caudal fins. However, *Gyrodactylus* sp. appeared to occur similarly on fish of all lengths, while *P. variegatus* tended to occur more abundantly on smaller rather than larger fish. At lower *Gyrodactylus* sp. infection levels (<100), the pelvic and pectoral fins were the main locations of attachment, followed by the dorsal fin. For infections of more than 100 parasites, more *Gyrodactylus* sp. were located on the pectoral fin. In another aspect, for a low number of *Paragyrodactylus variegatus* infections (<100), the pelvic and pectoral fins were the preferred locations of attachment, followed by the caudal fin. Between the months of April and September, there were many monogenean parasites on the fish fins, and the fish size was within the range of 5-10 cm. However, when a fish is greater than 1[illegible] long, the number of parasites on its fins is greatly reduced.

Key words: *Homatula variegate*; Gyrodactylidae; Site specificity; Qinling Mountains

Introduction

Among the members of the class Monogenea, viviparous gyrodactylids are one of the most common parasites in wild and cultured fish, causing great ecological and economic harm [1]. Some gyrodactylids show significant bit specificity, but this is highly variable among species. Some researchers are concerned with the site preference of *Gyrodactylus turnbulli* on *Poecilia reticulata* (guppy) in an

experimental environment. The study found that the tail pedicels (42%) and tail fins (40%) are more susceptible to parasitic infection [2].

However, the distribution of parasites on fish is strongly correlated with the age of infection. Other groups have reached similar conclusions by studying the behaviour of parasites(*G. colemanensis*) on the fry of *Salvelinus fontinalis* [2, 3]. Parasites attach to any part of the body's epithelial tissue. In particular, many parasites were found on the edges of the tail, pectoral and peritoneal fins.The parasite periodically migrates to the edges of the fins and can travel through the body to reach other fins [2, 4]. Recently, *Homatula variegata* (Dabry de Thiersant, 1874) became a fish of growing concern due to the potential aquaculture in China [5]. *Gyrodactylus* sp. (an apparently undescribed species) and *Paragyrodactylus variegatus* (You, King, Ye and Cone, 2014) [6] are two parasites that are found on the fins and, occasionally, the body surface, of *H. variegata* in Xunyangba, which is located by the Qinling Mountains in China (33.33°N, 108.33°E). However, gyrodactylids that live on the surface of fish appear to be less specific in terms of their environmental requirements and therefore occur in a variety of locations. This assumption, of course, has led to a lack of information on these forms of positioning; Most authors locate parasites on major branches of the body, such as the gills or torso [7]. However, no specific studies on the distribution of gyrodactylid parasites in *Homatula variegata* have been done. Therefore, this study attempts to describe the position specificity of *Gyrodactylus* in *Homatula variegata*.

Materials & Methods

Sample collection

Homatula variegata were collected with seine nets during late March to late September 2016 in Xunyangba (33.33°N, 108.33°E), Ningshan County located on the south aspects of the Qinling Mountains in Shaanxi Province, central China. The water temperature on the days of collection was recorded (Table 1).

Each fish was individually placed in a plastic tank filled with filtered river water and was transported to a field laboratory and examined within one hour. The fish were euthanized with excessive eugenol anaesthetic fluid and fixed with 5-10% formalin. The total length of each fish was recorded and the fins were examined for the presence of parasites which were removed and identified in temporary wet mounts immediately, then stored in formalin. Almost all the parasites stuck to the skin after immobilization. Voucher specimens of the parasites and host were deposited in the

Fish Disease Laboratory, Shaanxi Normal University (Accession number: *H. variegata*: HV20160012; *Gyrodactylus* sp.: GS20160001) and *P. variegatus*: PV20160001). The animal processing was authorized by the Animal Care and Use Committee of Shaanxi Normal University.

Statistical analysis

The parasite species and location of parasites on the host's fins were examined by using a two-way ANOVA, with the number of parasites on different fins used as the dependent variable. The microhabitat occurrence for each gyrodactylid species was determined by observing its position on the fins. The distributions of each gyrodactylid species on each of the different fins were compared by two-way ANOVA with multiple comparisons (Tukey's HSD test) to assess the significance of the difference. The significant level was set as $p < 0.05$. Pearson correlations were used to test for negative or positive associations between the density of the infections (mean number of parasites on each fin) of the two parasite species among fish fins. Finally, the relationships among the parasite density of infection, occurrences of different water temperature in the sampling location, and the number of parasites in fish fins and the fish body were assessed for the two parasite species using the Pearson correlations statistical analysis was conducted using SPSS (Statistical Package for Social Sciences, v21). We performed the Shapiro-Wilk test to assess the normality of the distribution.

Results

The number dynamics of gyrodactylid parasites

Temporal changes in the density of infection on the fins were analysed. The highest density of infection of *Gyrodactylus* sp. on fish fins appeared in April, while the highest density of infection of *P. variegatus* on fish fins occurred in March. The number of *Gyrodactylus* sp. and *P. variegatus* showed roughly similar trends, with the density of infection (Mean±SE) on fins decreasing in May (4.17±0.74 and 2.6±0.43, respectively). The density of infection on fins decreased in August (2.09±0.31 and 2.17±0.27) and rose in September (5.53±0.43 and 5.23±0.45) (Figure 1). Of all 7 months, the number dynamics of the two gyrodactylid parasites on the fins were lowest in August. There was no significant correlation between abundance and the water temperature, which ranged from 7ºC (in March) to 23ºC (in July) (*Gyrodactylus*

sp.: r=0.149, p=0.751; *P. variegatus*: r=0.090, p=0.847).

On the other hand, in Figure 2A, the number of *P. variegatus* on the pectoral fin was relatively large during May and July. The density of the infection of parasites detected on the dorsal fins and anal fins was relatively small. In Figure 2B, the number of parasites on different fins decreased significantly during April and May. In June, the number of *Gyrodactylus* sp. increased on the pectoral fin.

Distribution of parasites on fish fins

For *Gyrodactylus* sp., there was a significant difference (two-way ANOVA, F=11.97, df=4, p<0.001) among the mean number (mean ± SE) of parasites that were distributed on five fins. All data were examined from a total of 442 specimens of *Homatula variegata* from Xunyangba, Qinling Mountains of Shaanxi Province, central China, which were collected from March to September 2016. The total length of the host (±0.1 cm) ranges from 3.1 to 14.6 cm. The number of fish and the water temperature for each sampling period are recorded in Table 1. The mean number (mean ± SE) of *Gyrodactylus* sp. was higher on pectoral fins (2.73±0.252) and pelvic fins (2.36±0.218), and lower on the caudal fin (1.25±0.092) and the anal fin (1.36±0.122) (Table 2). For *P. variegatus*, there was also a significant difference (two-way ANOVA, F=30.94, df=4, p<0.001) among the mean numbers (mean ± SE) of parasites that were distributed on the five fins. The mean number (Mean ± SE) of *P. variegatus* on pectoral fins (1.17±0.134) and pelvic fins (0.97±0.075) was higher, and that on the lower in anal fin (0.41±0.040) and dorsal fin (0.37±0.046) was lower (Table 2).

In general, the mean number (Mean ± SE) of *Gyrodactylus* sp. infected on different fin parts was higher than that of *P. variegatus* (Table 2). However, we also detected a contrasting pattern between the densities of specific parasitic infections of fins. Although there is no difference between the pectoral fins and the pelvic fins of the two gyrodactylid parasites (Tukey's HSD, df = 4, p> 0.05), the parasite density of infection in these two fins was higher than of any other fin (Tukey's HSD, df =4, all p< 0.001; Table 2 and Figure 3). Patterns of parasite density of infection were comparable in *Gyrodactylus* sp. and *P. variegatus* but were clearly different from those on respective fins (two-way ANOVA, estimated marginal mean test).

By comparison, density of infection of *Gyrodactylus* sp. was significantly higheron pelvic fins and pectoral fins (test of between-subjects effects, F (4,4410), p< 0.001). Both parasite species showed intermediate density of infection in the dorsal fins

(*Gyrodactylus* sp.) and caudal fins (*P. variegatus*), respectively (Figure 3). On the other hand, the density of infection of *P. variegatus* was also significantly higher in pelvic, pectoral and caudal fins than that in anal and dorsal fins (Tukey's HSD, $df = 4$, both $p < 0.001$). No other significant differences in the density of specific parasitic infections between host fins were detected. At lower levels of infection, parasites preferentially colonized the pelvic, pectoral and dorsal fins. (Table 3). Most fins infect a small number of parasites. The number of *P. variegatus* present in fish is less than 100. As the intensity of each fish increases by 11 to 100 parasites, pelvic fins continue to be the main attachment, followed by the pectoral fin. However, at an intensity of 100 or higher, the parasite is concentrated on a specific number of fins. It was found that the number of infected fins did not increase as the total number of parasites increased. *Gyrodactylus* sp. is susceptible to pelvic fins. Obviously, there are dozens of parasites on some fins. However, for *P. variegatus*, the number of parasites on infected fins is low (Figure 5).

Relationships between the parasite density of infection on fins and the body length of fish

After statistical analysis, the number of *Gyrodactylus* sp. did not have a significant correlation(r=-0.1, p=0.059)with the body length of *H. variegata*. However, the number of *P. variegatus* is significantly negatively related to the body length of fish (r=-0.142, p<0.01) (Figure 4).

Of the 442 fish collected, 135 were only less than 7 cm in length, accounting for 30.5% of the total; 183 fish had a body length of more than 7 cm but less than 10 cm, accounting for 41.4% of the total;124 fish had a body length of more than 10 cm, accounting for 28.1% of the total. Interestingly, throughout the study period (March 2016 to September) host fish were infected with significant fluctuations in the two gyrodactylid parasites (Table 4). When the length of the fish exceeds 5 cm, the number of parasites on the fins is relatively high. However, the number of parasites on the fins will be greatly reduced as the length of the main body continues to increase.

Discussion

Although it has been reported in China, little research has been done on the survival of gyrodactylid on *Homatula variegata*, for which a total of two genera were found: *Gyrodactylus* (Nordmann, 1832) and *Paragyrodactylus* (Gvosdev et Martechov, 1953). Our investigation revealed that *Gyrodactylus* sp. and *P. variegatus* survive on this host.

The microhabitat of monogeneans living on fins has been investigated by many authors [8-10]. Monogeneans exhibit the characteristics of aggregate parasitism. For example, benedeniines are significantly parasitic on specific fins [8]. Studies have found that parasites that are attached to the dorsal fins or pelvic fins of fish may be designed to evade host predation, competition, and local immune responses [11,12]. In addition, each developmental cohort that inhabits different fish fins can receive exclusive food and spatial resources [10].

In the present study, the two species of parasites appear to have subtle spatial partitions in their common resources. *Gyrodactylus* sp. occurred most frequently on the pectoral and pelvic fins, while *P. variegatus* occurred on the pectoral fin, pelvic fin and caudal fins.

Fish sometimes lie down when they are underwater so that the fins are close to the ventral surface in a high-speed water environments [2]. Fish also often appear to compete for territory [2]. At light infections of gyrodactylid, parasites can be transferred between individual fish, using direct contact with the fins of the fish. However, at higher levels of parasitic infection, certain worms may fall off directly from the fish or leave freely [2]. In this study, we investigated the average water temperature of sampling points during sampling. Water temperature is thought to be a factor affecting the parasite's ability to reproduce [13-15]. It has been reported that some Monogenea parasite species have the longest life span at 21°C, and are not able to survive at 30°C [16]. Moreover, there is a certain degree of correlation between the water temperature and number dynamics of parasites on fins, but the influence of water temperature is distinct in different parasite species [17,18]. The relationship between the temperature and parasite reproduction is complex. Some literature has noted that the number dynamics increased with the rise of water temperature [19,20]. On the other hand, for some species, elevated temperature can be a limiting factor for survival and reproduction [21,22]. In our study, we found that the number dynamics of *Gyrodactylus* sp. and *P. variegatus* on the fins of *H. variegata* showed a different trend; specifically, the *Gyrodactylus* sp. density of infection on fins reached its highest point in April, gradually declined in summer and rose again in autumn (Figure 1). Some previous findings support the results of increased numbers in summer [16,23], which is consistent with our findings. In addition, although the water temperature in July(23°C) was higher than that in June(21°C), the volume dynamics of the two fin parasites in July were indeed lower than that in June. The reason may be the case that

the immunity of the host fish increases with the increase of the water temperature, thus leading to a decline in the number of parasites. Previous studies have demonstrated that the same results in higher levels of infection and weaker host immunity [17,18,24]. Another reason may be the change of water environmental factors that are caused by the increased water temperatures in July, which leads to a decrease in the number of water environmental factors. In addition to temperature, the photoperiod, salinity and water flow can influence the success of the infection. Studies have found that host fish are only infected by monogenean parasites during the day, and low-temperature and high-salt waters are more conducive to parasitic infections of fish [25]. Interestingly, the number of *Gyrodactylus* sp. on fish fins dropped significantly, relative to *P. variegatus*, in May. The cause for this was unknown at this time but could involve interspecific competition. The number of *Gyrodactylus* sp. rose again in September, possibly due to changes in the water flow rate. More detailed work on this topic is clearly required. We found there was a negative correlation between the number of *Gyrodactylus* sp. and the body length of *H. variegata*, and there was a relatively weak negative correlation between the number of *P. variegatus* and the body length of fish. This finding is similar to the results of some previous literature. In other words, there is a negative correlation between the richness of parasite species and the size of fish bodies [26-28]. Due to the increase in parasite abundance, smaller fish hosts may be more susceptible to disease than are large fish [1]. However, some previous literature observed the opposite, in which there was a positive correlation between the dynamics of the number of parasites on the fins and the size of the host [29-32]. Some researchers have pointed out that the relationship between the number of parasites in the infected sites of the fish and the length of the host should also be highlighted. This is more pronounced in small-sized fish, which have a higher density of parasitic infections on the body surface [33,34]. Another possible reason might be that fish with a longer body length may be found in microhabitats with less exposure to parasitic infections [28]. Fish use group behaviour and immune responses to reduce the risk of parasites [35]. Perhaps large fish are better suited to finding groups. The current scope of the study reveals that there is less aggregation of parasites in large *Homatula variegata* fins, which is consistent with the idea that the chances of avoiding infection are enhanced. It is useful to emphasize, however, that not only the host size but also the ecology of each host species affects the species richness of the parasite [32].

Different kinds of *Gyrodactylus* like to be parasitic in different parts of the host. *Gyrodactylus masu* is found on the body surface of salmonids, and the fins, gill arches and gill filaments are the main locations [2, 36]. By observing the parasitic behaviour of the five species of *Gyrodactylus* parasites, researchers found that four of the parasites like to parasitize on the surface and fish gills of fish. When studying the two parasites (*G. colemanensis* and *G. salmonis*) on the surface of the salmonids, it was found that most of the *G. colemanensis* were attached to the edge of the fin. *Gyrodactylus salmonis* like to attach to the head and body surface of the fish [2, 3]. Studies have found that different types of *Gyrodactylus* have different haptor shapes, which may lead to differences in their habitats [3,37]. The morphology of the haptors of each *Gyrodactylus* species is probably adapted to the surface of their host.

Conclusions

In summary, through the investigation of two parasites infecting *Homatula variegata*, we found that (1) the highest fin infection density of *Gyrodactylus* sp. appeared in April and March, respectively, whereas the highest density of the infection rate of *Paragyrodactylus variegatus* appeared in June. That is, the peak of relative abundance of the two parasites on the fins showed a time niche separation. However, the trend of number dynamics on fins is similar during the period from May to September. The number of the two parasites on the fins of the host rose again in the autumn. (2) The two gyrodactylid parasites seem to partition their common resources spatially. *Gyrodactylus* sp. preferred to parasitize on the pectoral and pelvic fins, while *P. variegatus* preferred to parasitize on the pectoral, pelvic and caudal fins. This may be explained by the avoidance of predation competition and the host's local immune response. This bit selection mode can be used as a potential delivery policy in Gyrodactylids. The main factors leading to the adhesion of specific habitats have not yet been determined and may be linked to physiological, environmental, ecological and physical factors. More research is required to clarify this.

Acknowledgements

The authors sincerely thank Fenhong Li, Fei Ye, Jun Yan, Junqing Jia and Xue Lan (College of Life Sciences, Shaanxi Normal University) for capturing experimental samples and collecting data, and Dr. David K. Cone (Department of Biology, Saint

Mary's University, Canada) and Fei Ye for his valuable suggestions for the manuscript. Our research has been funded by the National Natural Science Foundation of China (31872203) and the Natural Science Foundation of Shaanxi Province (2017JM3014).

Author contributions

Formal analysis: Xiaoning Chen.

Funding acquisition: Ping You.

Investigation: Xiaoning Chen, Biao Wang, Jianzhen Nie, Ping You.

Methodology: Xiaoning Chen.

Project administration: Ping You.

Resources: Xiaoning Chen, Biao Wang, Jianzhen Nie.

Software: Xiaoning Chen.

Supervision: Ping You.

Writing - original draft: Xiaoning Chen.

Writing - review & editing: Ping You.

References

1. Kate SH, Alexander KB, David BV, Alejandro TG. Monogenean parasite cultures: current techniques and recent advances. Advances in Parasitology, 2018; 99: 61-91. https://doi.org/10.1016/bs.apar.2018.01.002

2. Jenson AJ, Johnsen BO. Site specificity of Gyrodactylus salaris Malmberg, 1957 (Monogenea) on Atlantic salmon (Salmo salar L.) in the River Lakselva, northern Norway. Canadian Journal of Zoology, 2011; 70(2): 264-267. https://doi.org/10.1139/z92-039

3. Cone DK, Cusack R. A study of *Gyrodactylus colemanensis* Mizelle and Kritsky, 1967 and *Gyrodactylus salmonis* (Yin and Sproston, 1948) (Monogenea) parasitizing captive salmonids in Nova Scotia. Canadian Journal of Zoology, 1988; 66: 409-415.

4. Johnsen BO, Jensen AJ. Infection of Atlantic salmon, *Salmo salar* L., by *Gyrodactylus salaris*, Malmberg 1957, in the River Lakselva, Misvœr in northern Norway. Journal of Fish Biology, 1992; 40: 433-444.

5. Zhou XK, Zhu CY, He HB, Wang XL, Xu HM, Wu JY, Wen AX. Analysis of

nutritional components and safety evaluation for the muscles of Paracobitis variegates. Freshwater Fisheries, 2018; 48(2), 87-93.

6. You P, King SD, Ye F, Cone DK. *Paragyrodactylus variegatus* n. sp. (Gyrodactylidae) from *Homatula variegata* (Dabry De Thiersant, 1874) (Nemacheilidae) in central China. *Journal of Parasitology*, 2014; 100(3): 350-355. https://doi.org/10.1645/13-257.1
7. Rameshkumar G, Ravichandran S, Ramesh M. Distribution of isopod parasites in Carangid fishes from *Parangipettai*, Southeast coast of India. *Journal of Parasitic Diseases*, 2014; 40(1): 124-128.
8. Whittington ID, Horton MA. A revision of *Neobenedenia* Yamaguti, 1963 (Monogenea: Capsalidae) including a redescription of *N. melleni* (MacCallum, 1927) Yamaguti, 1963. Journal of Natural History, 1996; 30: 1113-1156.
9. Hirazawa N, Hagiwara H, Takano R, Noguchi M, Narita M. Assessment of acquired protection levels against the parasite *Neobenedenia girellae* (Monogenea) between body surface sites including fins of amberjack *Serioladumerili* (Carangidae) and the skin in response to the parasite infection. *Aquaculture*, 2011; 310(3-4): 252-258. https://doi.org/10.1016/j.aquaculture.2010.10.029
10. Alejandro TG, Constantin CC, Richard R, Kate SH. Tracking transparent monogenean parasites on fish from infection to maturity. *International Journal for Parasitology: Parasites and Wildlife*, 2015; 4: 316-322. https://doi.org/10.1016/j.ij-ppaw.2015.06.002
11. Jones SRM. The occurrence and mechanisms of innate immunity against parasites in fish. *Developmental & Comparative Immunology*, 2001; 25(8-9): 841-852. https://doi.org/10.1016/S0145-305X(01)00039-8
12. Whittington ID, Ernst I. Migration, site-specificity and development of *Benedenia lutjani* (Monogenea: Capsalidae) on the surface of its host, *Lutjanus carponotatus* (Pisces: Lutjanidae). *Parasitology*, 2002; 124: 423-434.
13. Chubb JC. Seasonal occurrence of helminthes in fresh water fishes. Part Ⅰ. Monogenea. *Advances in Parasitology*, 1977; 15: 133-199.
14. Bauer ON, Karimov SB. Patterns of parasitic infections of fishes in a water body with constant temperature. *Journal of Fish Biology*, 1990; 36(1): 1-8.
15. Özer A, Kornyychuk Y, Öztürk T, Yurakhno V. Levels of infection and seasonal occurrence of *Gyrodactylus alviga* (Monogenea: Gyrodactylidae) on the whiting

Merlangius merlangus off the Turkish and Russian coasts of the Black Sea. *Journal of Helminthology*, 2016; 90(3): 347-352. https://doi.org/10.1017/S0022149X1500036X

16. Jansen PA, Bakke TA. (1991). Temperature-dependent reproduction and survival of *Gyrodactylus salaris* Malmberg, 1957 (Platyhelminthes: Monogenea) on the Atlantic salmon (*Salmo salar* L.). *Parasitology*, 1991; 102(1): 105-112. https://doi.org/10.1017/S0031182000060406
17. Mo TA. Seasonal occurrence of *Gyrodactylus derjavini* (Monogenea) on brown trout, *Salmo trutta*, and Atlantic salmon, *S. salar*, in the Sandvikselva river, Norway. *Journal of Parasitology*, 1997; 83(6): 1025-1029. https://doi.org/10.2307/3284356
18. Andersen PS, Buchmann K. Temperature dependent population growth of *Gyrodactylusderjavini* on rainbow trout, *Oncorhynchus mykiss*. *Journal of Helminthology*, 1998; 72(1): 9-14. https://doi.org/10.1017/S0022149X00000900
19. Appleby C. Population dynamics of *Gyrodactylus*sp. (Monogenea) infecting the sand goby in the Oslo Fjord, Norway. *Journal of Fish Biology*, 1996; 49(3): 402-410.
20. Appleby C, Mo TA. Population dynamics of *Gyrodactylussalaris* (Monogenea) infecting Atlantic salmon, *Salmo salar*, parr in the river Batnfjordselva, Norway. Journal of Parasitology, 1997; 81(1): 23-30. https://doi.org/10.2307/3284312
21. Gonzalez-Lanza CM, Alvarez-Pellitero PM. Description and population dynamics of *Dactylogyrus legionensis* n. sp. From *Barbus barbus bocagei* Steind. *Journal of Helminthology*, 1982; 56(3): 263-273.
22. Koskivaara M, Valtonen ET. (1992). *Dactylogyrus* (Monogenea) communities on the gills of roach in three lakes in Central Finland. *Parasitology*, 1992; 104(2): 263-272.
23. Mo TA. Seasonal variations in the prevalence and infection intensity of *Gyrodactylus salaris* Malmberg, 1957 (Monogenea: Gyrodactylidae) on Atlantic salmon parr, *Salmo salar* L. in the River Batnfjordselva. *Journal of Fish Biology*, 1992; 41(5): 697-707.
24. Kim KH, Kyoung JA, Chun SK. Seasonal abundances of *Prosomicrocotylagotoi*(Monogenea) and *Opecoelussphaericus* (Digenea) from greenlings *Hexagram mosotakii* in a southern coastal area in Korea. *Aquaculture*, 2001; 192(2): 147-153.

25. Brazenor AK, Hutson KS. Effects of temperature and salinity on the life cycle of *Neobenedenia* sp. (Monogenea: Capsalidae) infecting farmed barramundi (Latescalcarifer). *Parasitology Research*, 2015; 114: 1875–1886.
26. Somdal O, Schram TA. (1992). Ectoparasites on northeast Atlantic mackerel (*Scomber scombrus* L.) from western and North Sea stocks. *Sarsia*, 1992; 77(1): 19-31.
27. Combes C, Euzet L. What makes a fish a suitable host for Monogenea in the Mediterranean. *International Journal for Parasitology*, 1997; 71(3): 203-210.
28. Hayward CJ, Perera KML, Rohde K. Assemblages of ectoparasites of a pelagic fish, slimy mackerel (*Scomber australasicus*), from south-eastern Australia. *International Journal for Parasitology*, 1998; 28(2): 263-273. https://doi.org/10.1016/S0020-7519(97)00186-0
29. Khidr AA. Population dynamics of *Enterogyrus cichlidarum* (Monogenea: Ancyrocephalinae) from the stomach of *Tilapia* spp. in Egypt. *International Journal for Parasitology*, 1990; 20(6): 741-745. https://doi.org/10.1016/0020-7519(90)90007-A
30. Rohde K, Hayward C, Heap M. Aspects of the ecology of metazoan ectoparasites of marine fishes. *International Journal for Parasitology*, 1995; 25(8): 945-970. https://doi.org/10.1016/0020-7519(95)00015-T
31. Morand S, Poulin R, Rohde K, Hayward C. Aggregation and species coexistence of ectoparasites of marine fishes. *International Journal for Parasitology*, 1999; 29(5): 663-672. https://doi.org/10.1016/S0020-7519(99)00029-6
32. Matejusova I, Morand S, Gelnar M. Nestedness in assemblages of Gyrodactylids (Monogenea: Gyrodactylidea) parasitising two species of cyprinid-with reference to generalists and specialists. *International Journal for Parasitology*, 2000; 30(11): 1153-1158. https://doi.org/10.1016/S0020-7519(00)00097-7
33. George-Nascimento M, Muñoz G, Marquet PA, Poulin P. (2004). Testing the energetic equivalence rule with helminth endoparasites of vertebrates. *Ecology Letters*, 2004; 7: 527–531.
34. Poulin R, George-Nascimento M. The scaling of total parasite biomass with host body mass. *International Journal for Parasitology*, 2007; 37(3-4): 359–364. https://doi.org/10.1016/j.ijpara.2006.11.009
35. Mikheev VN, Pasternak AF, Taskinen J, Valtonen TE. Grouping facilitates avoidance of parasites by fish. *Parasites & Vectors*, 2013; 6: 301.

https://doi.org/10.1186/1756-3305-6-301

36. Ogawa K. A monogenean parasite *Gyrodactylusmasu* sp. n. (Monogenea: Gyrodactylidae) of salmonid fish in Japan. *The Bulletin of the Japanese Society of Scientific Fisheries*, 1986; 52: 947-950.

37. Al-Zubaidy AB. First Record of three monogenic parasites species from Iraqian freshwater fishes. *JKAU: Marine Science*, 2007; 18: 83-94.

Tables and Figures

Table 1. Morphometrics of *Homatula variegata* collected.

Month	Water temperature (℃)	Number of Fish	Body length (cm)	Average body length Mean ± SE（cm)
March	7	43	3.60-13.60	5.70±0.372
April	13	40	3.80-13.80	8.43±0.425
May	17.5	42	4.10-11.70	7.15±0.303
June	21	46	4.50-14.60	8.37±0.370
July	23	85	4.5-14.00	9.10±0.211
August	17	100	3.10-13.90	9.36±0.218
September	15	86	3.40-13.80	7.91±0.287

Table 2. The mean number (±SE) of two parasite species on different fish fin of *Homatula variegata*.

	Caudal fin	Anal fin	Dorsal fin	Pelvic fin	Pectoral fin
Gyrodactylus sp.	1.25±0.092	1.36±0.122	2.04±0.189	2.36±0.218	2.73±0.252
Paragyrodactylus variegatus	0.96±0.073	0.41±0.040	0.37±0.046	0.97±0.075	1.17±0.080

Table 3. Site specificity of *Gyrodactylus* sp. and *Paragyrodactylus variegatus* at four different range of infection of *Homatula variegata* (The average number of infections is on each fin).

Gyrodactylus sp.							*Paragyrodactylus variegatus*						
Nop	Nof	Cau	Ana	Dor	Pel	Pec	Nop	Nof	Cau	Ana	Dor	Pel	Pec
0	53	0	0	0	0	0	0	79	0	0	0	0	0
1~10	271	0.727	0.539	0.738	0.86	1.251	1~10	331	0.925	0.378	0.347	0.876	1.187
11~100	114	3.07	3.693	5.579	6.342	6.781	11~100	35	3.457	1.571	1.657	4.057	3.657
101~200	3	3	16	29	36.667	41.667	101~200	0	N/A	N/A	N/A	N/A	N/A

Note: Nop: Range in parasites Number; Nof: Number of Fish; Cau: Caudal fin; Ana: Anal fin; Dor: Dorsal fin; Pel: Pelvic fin; Pec: Pectoral fin.

Table 4. Parasitism by *Gyrodactylus* sp. and *Paragyrodactylus variegatus* on *Homatula variegata*.

Gyrodactylus sp.								*Paragyrodactylus variegatus*							
Month	Fs (cm)	Nof	Cau	Ana	Dor	Pel	Pec	Month	Fs	Nof	Cau	Ana	Dor	Pel	Pec
March 2016	1-5	25	85	97	125	134	68	March 2016	1-5	25	22	6	20	20	12
	5-10	14	68	104	102	118	134		5-10	14	31	19	28	47	23
	10-15	4	6	28	51	90	90		10-15	4	20	6	12	16	16
April 2016	1-5	7	9	16	31	32	29	April 2016	1-5	7	0	0	0	2	2
	5-10	19	48	71	145	168	192		5-10	19	7	9	13	12	13
	10-15	14	10	71	141	125	184		10-15	14	2	12	5	22	15
May 2016	1-5	8	9	12	19	15	12	May 2016	1-5	8	2	3	1	4	6
	5-10	32	23	17	22	20	23		5-10	32	29	9	8	26	22
	10-15	3	2	0	1	1	1		10-15	3	0	0	0	1	0
June 2016	1-5	6	8	2	9	3	29	June 2016	1-5	6	11	1	2	1	13
	5-10	31	67	40	45	77	116		5-10	31	46	20	9	54	98
	10-15	10	9	3	11	15	21		10-15	10	7	2	2	11	15
July 2016	1-5	1	0	0	1	1	0	July 2016	1-5	1	2	3	1	0	0
	5-10	57	52	40	52	52	85		5-10	57	31	14	14	25	67
	10-15	27	19	18	23	24	30		10-15	27	16	7	5	17	23
Aug. 2016	1-5	4	1	0	0	0	0	Aug. 2016	1-5	4	0	0	0	0	3
	5-10	57	29	15	19	31	52		5-10	57	27	6	8	35	82
	10-15	39	16	7	21	9	8		10-15	39	15	5	3	13	20
Sep. 2016	1-5	19	27	11	13	24	32	Sep. 2016	1-5	19	60	11	6	17	8
	5-10	47	54	45	42	66	86		5-10	47	84	34	19	77	51
	10-15	20	10	4	29	16	17		10-15	20	11	14	3	7	16

Note: Fs: Range in fish body size; Nof: Number of Fish; Cau: Caudal fin; Ana: Anal fin; Dor: Dorsal fin; Pel: Pelvic fin; Pecf: Pectoral fin.

Figure 1. Number dynamics (density of infection, Mean±SE) of *Gyrodactylus* sp. and *Paragyrodactylus variegatus* on different fish fins by month.

Figure 2. Parasites number dynamics (density of infection, Mean±SE) on different fish fin with monthly. A. *Gyrodactylus* sp., B. *Paragyrodactylus variegatus,* the abbreviation Ana., Cau., Dor., Pec., Pel., indicate, Anal fin, Caudal fin, Dorsal fin, Pectoral fin and Pelvic fin, respectively.

Figure 3. Number of *Gyrodactylus* sp. and *Paragyrodactylus variegatus* at different fish fin, the abbreviation Ana., Cau., Dor., Pec., Pel., indicate, Anal fin, Caudal fin, Dorsal fin, Pectoral fin and Pelvic fin, respectively. *$p < 0.05$; **$p < 0.01$.

Figure 4. Relationships between number of parasites on fish fins and body length of fish, *$p < 0.05$; **$p < 0.01$, A. *Gyrodactylus* sp., B. *Paragyrodactylus variegatus*.

Figure 5. Site specificity of *Gyrodactylus* sp. (A) and *Paragyrodactylus variegatus* (B) at increasing intensities of infection (from 1 to 100) of *Homatula variegate*.

Figure 1

Click here to access/download;Figure;Figure 1.tif

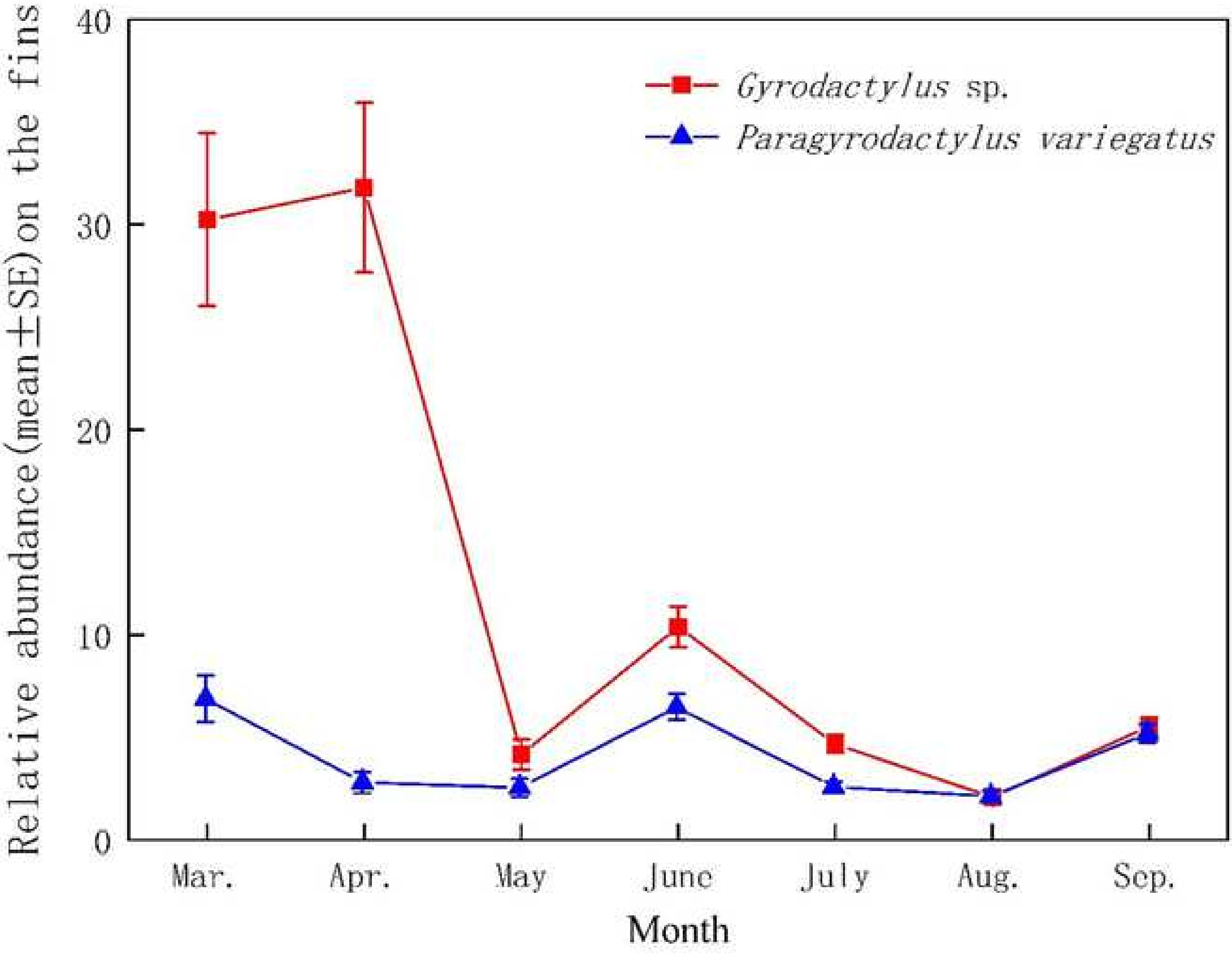

Figure 2

Click here to access/download;Figure;Figure 2.tif

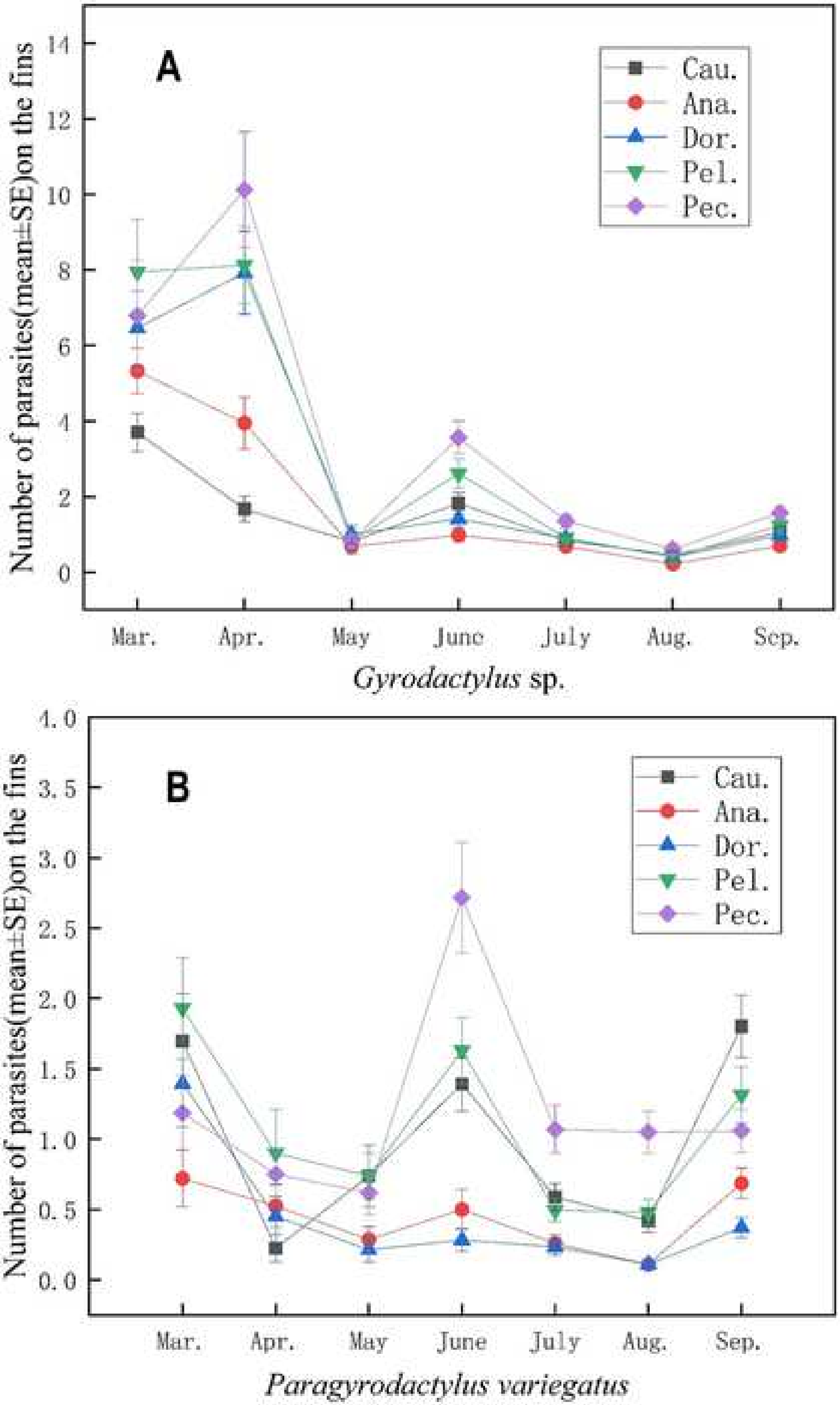

Figure 3

Click here to access/download;Figure;Figure 3.tif

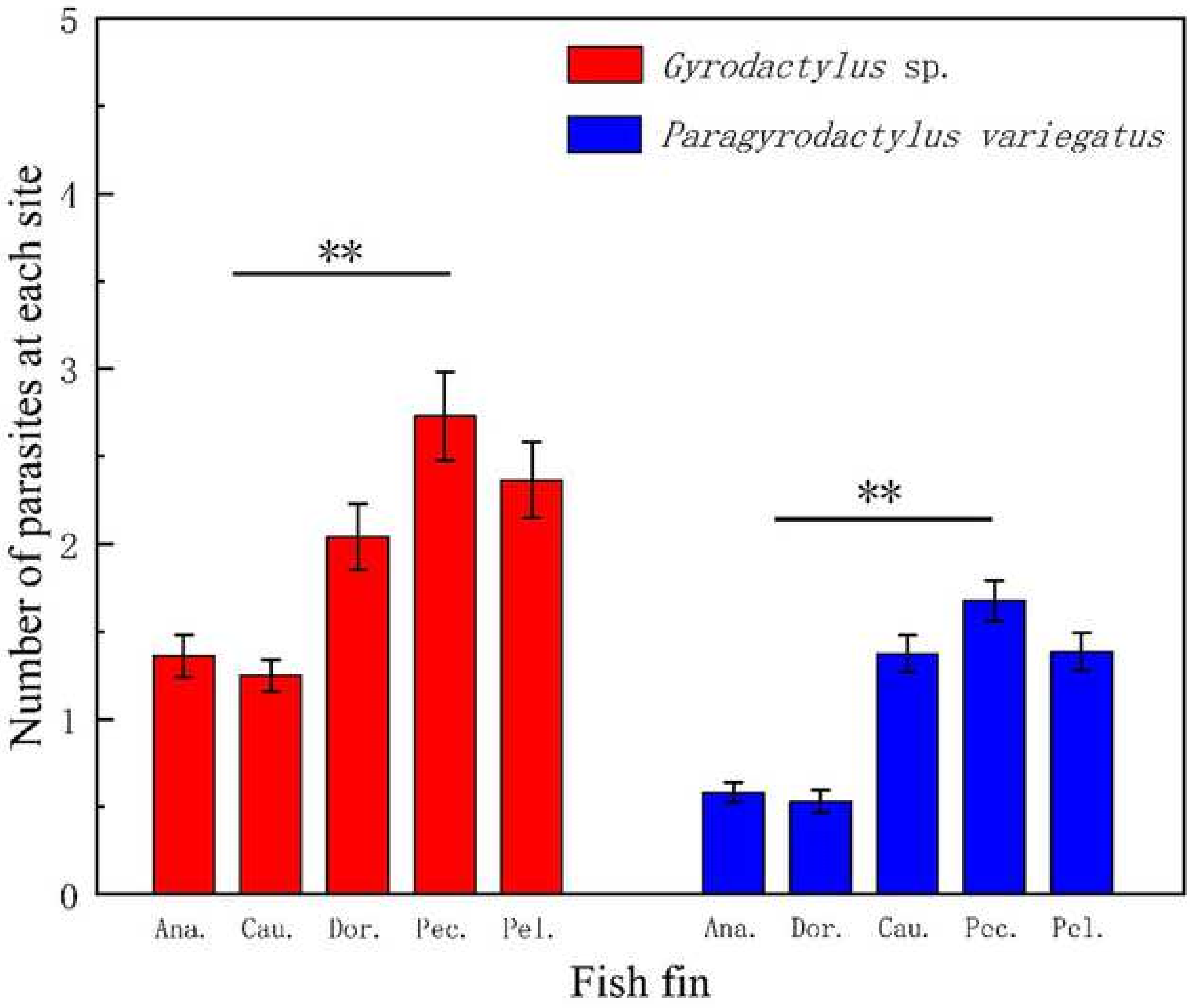

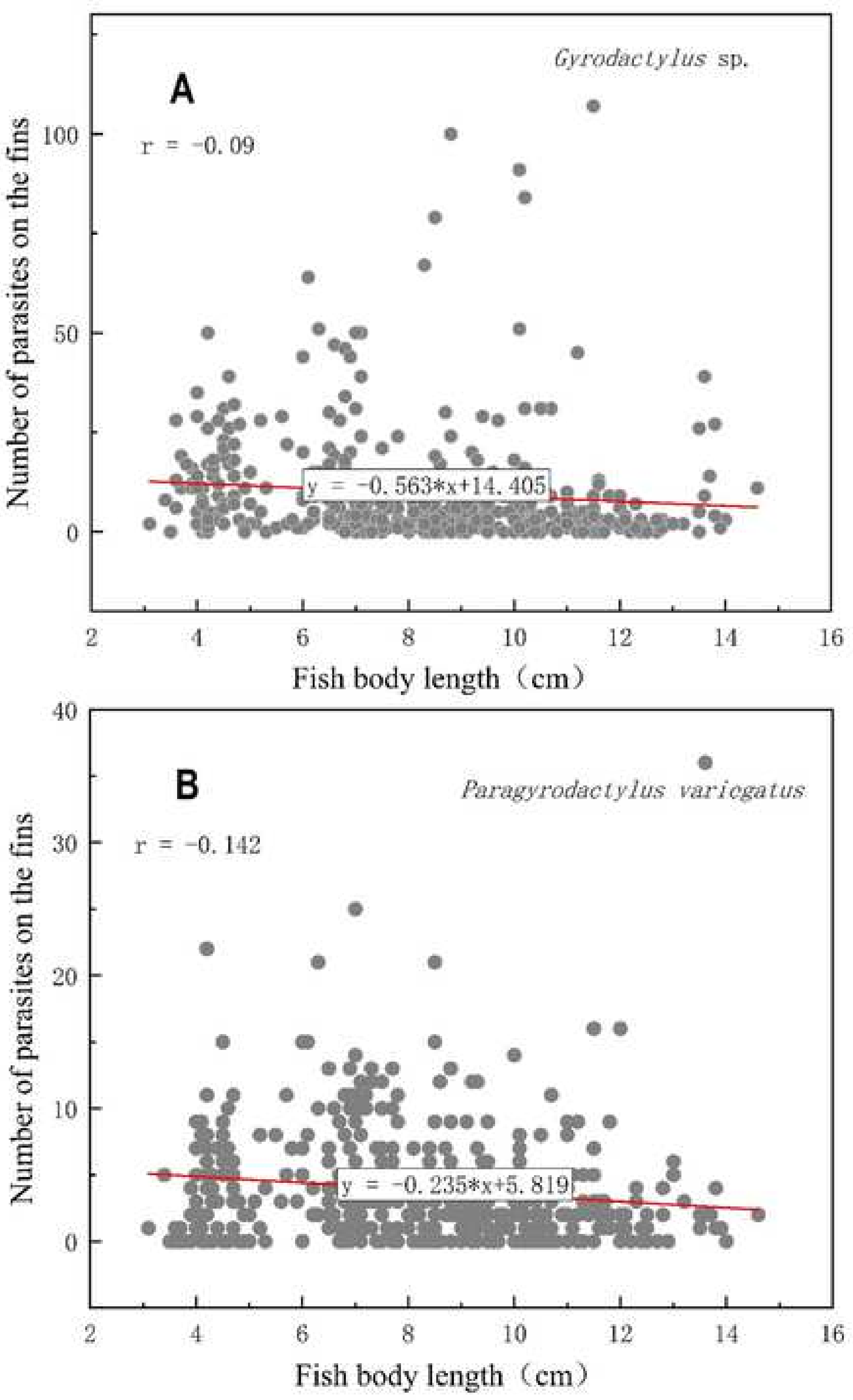
A
Gyrodactylus sp.
r = -0.09
y = -0.563*x+14.405
Number of parasites on the fins
Fish body length (cm)
B
Paragyrodactylus variegatus
r = -0.142
y = -0.235*x+5.819
Number of parasites on the fins
Fish body length (cm)

Figure 5

Click here to access/download;Figure;Figure 5.tif

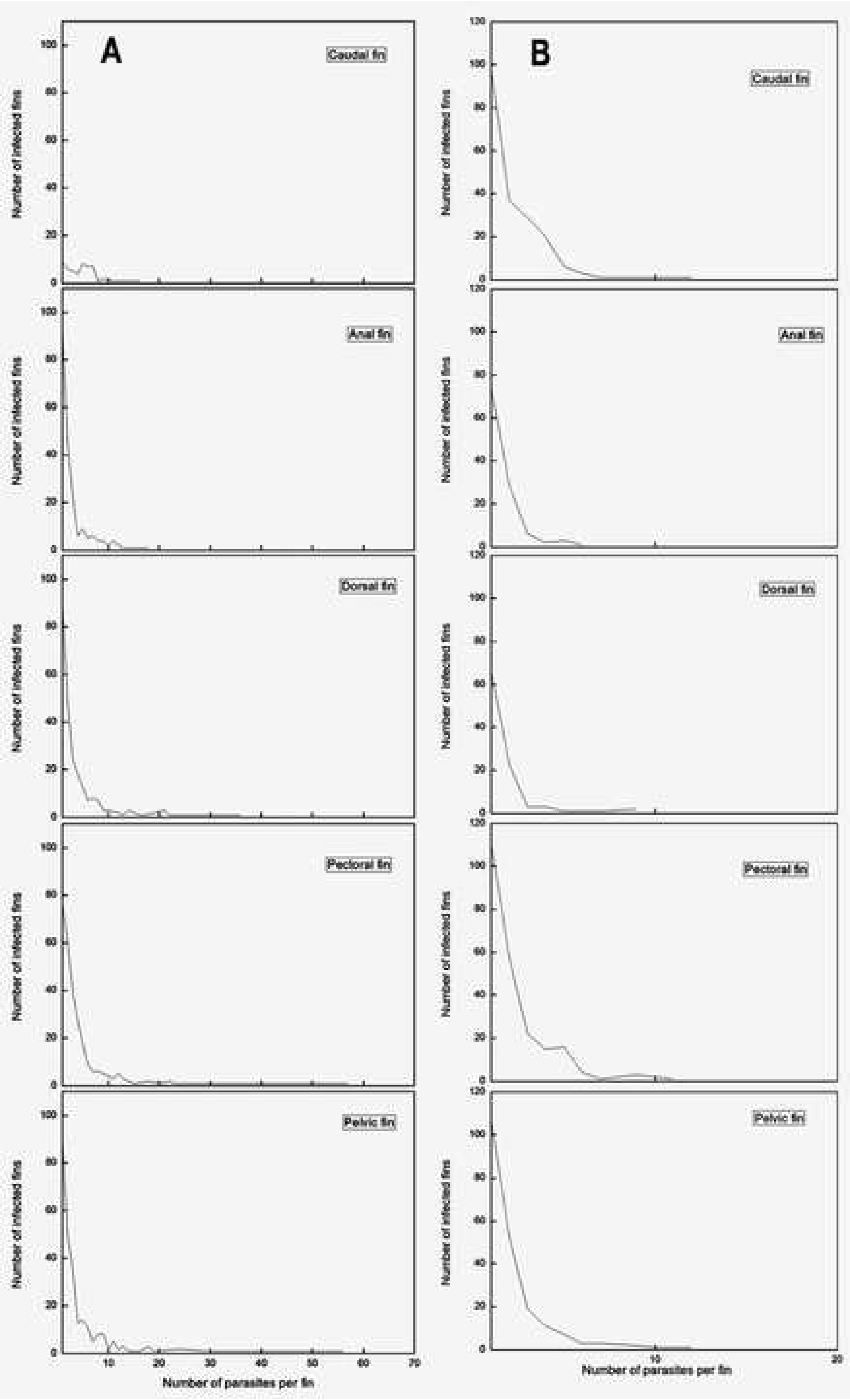

Table S1 Data of the gyrodactylids infecting Homatula variegata in March to September 2016

Click here to access/download
Supporting Information
Raw Data Chen-S1.xlsx
